# Triad of Diabetic Ketoacidosis, Hypertriglyceridemia, and Acute Pancreatitis: Severity of Acute Pancreatitis May Correlate with the Level of Hypertriglyceridemia

**DOI:** 10.7759/cureus.4930

**Published:** 2019-06-18

**Authors:** Sanjay Timilsina, Sambida Timilsina, Amrendra Mandal, Rabin Paudel, Vijay Gayam

**Affiliations:** 1 Internal Medicine, Interfaith Medical Centre, Brooklyn, USA; 2 Internal Medicine, Interfaith Medical Center, Brooklyn, USA

**Keywords:** diabetic ketoacidosis, acute pancreatitis, hypertriglyceridemia

## Abstract

A 50-year-old African-American male with no known previous medical comorbidities presented to the emergency room with complaints of three days of worsening epigastric pain associated with nausea and vomiting. Laboratory parameters on admission revealed high lipase: 1796 U/L (normal range 0-160 U/L), high blood glucose level: 300 mg/dl, anion gap metabolic acidosis, ketonuria, significant hyperlipidemia (triglyceride: 1226 mg/dl (normal range <150 mg/dl), and LDL cholesterol: 307 mg/dl (normal range <100 mg/dl)). Treatment with intravascular volume and electrolytes replacement as well as administration of intravenous insulin successfully resolved diabetic ketoacidosis (DKA) and hypertriglyceridemia (HTG) with a drop in triglyceride (TG) level from 1226 mg/dl to 193 mg/dl. Radiologic imaging studies by ultrasonography (USG) and CT of the abdomen showed features suggestive of interstitial pancreatitis. Glycated hemoglobin (HbA1) was 10.7% suggesting uncontrolled diabetes mellitus. Here, we explain the possible pathophysiology and management of this uncommon triad-DKA, hypertriglyceridemia, and acute pancreatitis-followed by discussion and literature review, which highlight the diagnostic challenge and possible relation of the severity of pancreatitis with the degree of hypertriglyceridemia.

## Introduction

Hypertriglyceridemia (HTG) is an important cause of acute pancreatitis (AP) and is considered a significant risk when levels are >1000 mg/dl [1]. The pathogenesis is explained by the breakdown of triglycerides (TGs) into toxic free fatty acids (FFA) by pancreatic lipase that causes lipotoxicity. The marked elevation of serum triglycerides occurs during episodes of diabetic ketoacidosis (DKA). The triad of DKA, AP, and hypertriglyceridemia is rare to find and is fatal in its disease course. Suspicion should be held high for AP in a patient with DKA and HTG since the clinical findings along with marked elevation of amylase and lipase in DKA can mask AP. The diagnosis of AP should be based on typical clinical manifestations, elevated amylase or lipase and confirmed with imaging. Our case describes a 50-year-old male patient who presented with mild acute pancreatitis, hypertriglyceridemia (TG level: 1226 mg/dl) and DKA and was a previously undiagnosed diabetic. This article presents the hospital course and management and discusses the case on the basis of the case findings and literature review.

## Case presentation

A 50-year-old African-American male with no significant comorbidities and not on any medications presented to the emergency department with complaints of three days of worsening epigastric pain. The pain was dull, non-radiating and 10/10 in intensity, associated with nausea and three episodes of vomiting. There was no history of similar pain episodes in the past. He was a chronic smoker and consumed a bottle of beer a few days in a week with the last drink six days prior to presentation. On examination, the patient was found to have a BMI of 29. He was in distress due to pain (10/10) but was alert and oriented. The patient was tachycardic with a heart rate of 102 beats/minute; other vitals were stable. An abdominal examination revealed epigastric tenderness with no rebound tenderness and negative Murphy's sign. Bowel sounds were sluggish with no palpable organomegaly or lumps. Cardiovascular and respiratory system examination revealed no abnormalities.

**Figure 1:**
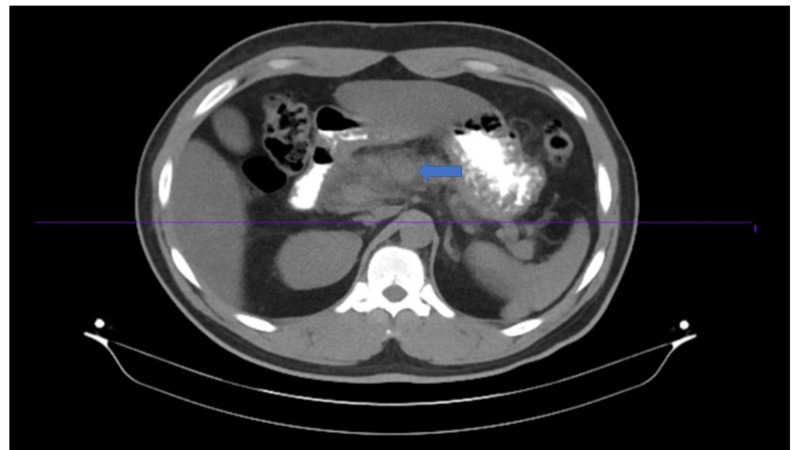
Cross-sectional image of CT abdomen with blue arrow showing the inflammation surrounding the pancreas compatible with acute pancreatitis.

**Figure 2:**
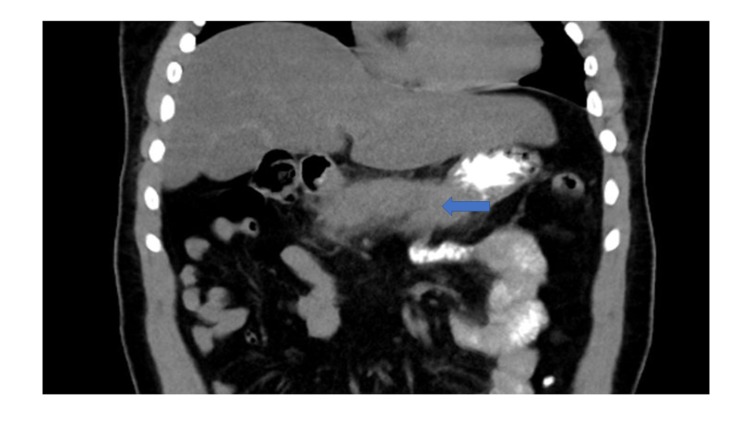
Coronal sectional image of CT abdomen with blue arrow showing the inflammation surrounding the pancreas compatible with acute pancreatitis.

The patient was kept nil by mouth, and aggressive intravenous fluids were administered. Morphine was given for pain. Investigations were ordered in the line of the acute abdomen. Lipase was high: 1796 U/L (normal range 0-160 U/L). Other significant lab findings were leukocytosis of 12,800 per cu mm (normal range:4500-1100 per cu mm), hematocrit of 47% (normal range 40-54%), and blood glucose of 300 mg/dl. Arterial blood gas (ABG) analysis showed anion gap metabolic acidosis with a pH of 7.1 (normal pH:7.35-7.45), HCO3 of 8.3 mEq/L (normal range 22-28 mEq/L), and PCO2 of 20 mm Hg on room air (normal range: 38-42 mm Hg). Besides sodium (Na) of 132 mEq/L (normal range 135- 145 mEq/L), all other electrolytes were normal. HBA1c was 10.7% (normal range: 4-5.6%). All these metabolic abnormalities were questionable given the mild presentation of acute pancreatitis. However, urinalysis showed ketonuria and glycosuria. Based on lab parameters and ABG analysis, a diagnosis of DKA was made. He was transferred to the ICU for closer monitoring. Insulin infusion and IV fluid were started. Meanwhile, imaging finding by ultrasonography (USG) abdomen showed inflamed pancreas and no gallstones. CT of the abdomen (Figures 1-2) showed inflammation surrounding the pancreas compatible with acute pancreatitis. No focal fluid collection was seen. No other acute abnormalities were observed in the abdomen or pelvis. Lipid profile showed a high TG level of 1226 mg/dl and a high LDL cholesterol of 307 U/L. TG level declined from 1226 mg/dl to 193 mg/dl after treatment with IV insulin in two days. After the closure of the anion gap and when the patient was able to tolerate feeding, diet was started and gradually advanced as tolerated. Anti GAD 65 Ab was tested, which was negative, favoring type 2 diabetes. The patient was discharged on bolus and basal insulin and atorvastatin.

## Discussion

There are very few reports of literature on combined presentation of DKA, hypertriglyceridemia, and acute pancreatitis. Poorly controlled diabetes mellitus can trigger hypertriglyceridemia. The pathophysiology is lack of insulin that results in increased production of very-low-density lipoprotein (VLDL) from the liver because of increased lipolysis, release and delivery of FFAs to the liver coupled with the inhibition of lipoprotein lipase in the peripheral tissues. The marked elevation of serum triglycerides occurs during episodes of DKA and HTG is considered a significant risk for acute pancreatitis when levels are >1000 mg/dl. On the other hand, DKA can also occur as a complication of acute pancreatitis in diabetic patients. But pancreatitis as a cause of DKA is less likely in this patient, given the mild presentation of pancreatitis. The systemic complications are uncommon and much less severe in patients with interstitial pancreatitis [2].

DKA is a fatal manifestation of diabetes, the prevalence of which is increasing [3]. It can be the first presentation of a diabetic as in our patient who presented with acute abdomen. The clinical challenge lies in diagnosing DKA in the presence of acute pancreatitis and vice-versa. Each of the enzymes amylase and lipase are elevated in patients with DKA, which can be threefold or higher. Moreover, frequently, serum amylase can be normal in patients with acute pancreatitis secondary to hyperlipidemia due to the suppression or inhibition of serum amylase activity in such patients [4]. In addition, the diagnosis of pancreatitis in patients with DKA should be primarily based on the clinical findings and imaging [5]. Ultrasonography (USG) is a less expensive, easily available and effective option in the initial instance to exclude gall stone pancreatitis. We would also like to highlight the importance of initial lipid profiling in patients with DKA to prevent delays in diagnosing the triad of DKA, hypertriglyceridemia, and acute pancreatitis, which can be potentially fatal.

Our patient presented with mild acute pancreatitis. Upon reviewing other similar case reports on this triad, we noticed a milder presentation in patients with lower level of TG and severe presentation in patients with very high TG level [6-9]. There has also been a retrospective study on a large number of patients who depicted a more severe form of pancreatitis in the higher TG groups than that in the lower TG groups implying that an elevated TG level may be associated with a poor prognosis [10]. Our case findings were in support of this study. In our case, intravascular volume, electrolyte replenishment, and insulin infusion resulted in the correction of DKA as well as HTG. HTG dropped down to 193 mg/dl from 1276 mg/dl (normal range: <150 mg/dl) in two days.

## Conclusions

Physicians need to be aware of the importance of lipid panel and early abdominal imaging in a patient presenting with DKA coupled with elevated serum amylase or lipase to make an early diagnosis of acute pancreatitis and to initiate timely management of this clinical triad to prevent morbidity and mortality.
